# Impact of Body Mass Index on the Age of Relapsing-Remitting Multiple Sclerosis Onset: A Retrospective Study

**DOI:** 10.3390/neurolint13040051

**Published:** 2021-10-11

**Authors:** Vasileios Siokas, Konstantinos Katsiardanis, Athina-Maria Aloizou, Christos Bakirtzis, Ioannis Liampas, Evangelos Koutlas, Jobst Rudolf, Konstantinos Ntinoulis, Jannis Kountouras, Efthimios Dardiotis, Georgia Deretzi

**Affiliations:** 1 Laboratory of Neurogenetics, Department of Neurology, University Hospital of Larissa, University of Thessaly, 41110 Larissa, Greece; vsiokas@med.uth.gr (V.S.); aaloizou@med.uth.gr (A.-M.A.); liampasioannes@gmail.com (I.L.); 2 Department of Neurology, Papageorgiou General Hospital, 56403 Thessaloniki, Greece; dinosgr@gmail.com (K.K.); koutlasevangelos@gmail.com (E.K.); jobstrudolf@hotmail.com (J.R.); kostas.ntinoulis@gmail.com (K.N.); gderetzi@gmail.com (G.D.); 3 B’ Department of Neurology, AHEPA University Hospital, Aristotle University of Thessaloniki, 54636 Thessaloniki, Greece; bakirtzischristos@yahoo.gr; 4 Department of Medicine, Second Medical Clinic, Ippokration Hospital, Aristotle University of Thessaloniki, 54642 Thessaloniki, Greece; jannis@auth.gr

**Keywords:** body mass index, age at onset, metabolic syndrome, RRMS

## Abstract

A BACKROUND: Multiple sclerosis (MS) is a complex chronic disease of the central nervous system (CNS). Body mass index (BMI), a component of metabolic syndrome (MetS), is considered among the risk factors for MS. However, its role in MS remains ambiguous. OBJECTIVE: To examine the impact of BMI on the age of onset in patients with relapsing-remitting MS (RRMS) in a Greek cohort. METHODS: Data from 821 Greek patients with RRMS were collected. The BMI values were considered as quartiles. Comparisons for the demographic characteristics between the quartiles were made by Pearson’s chi-square test for the categorical variables and by ANOVA for the continuous variables. An overall p-value was calculated corresponding to trend for association. In case of significant association, further post-hoc analysis was performed in order to identify differences in demographic characteristics between specific BMI quartiles groups. Linear regression analyses were used to assess the relationship between BMI and age at onset of MS. RESULTS: Comparisons of participant characteristics by quartiles of BMI revealed that participants with the highest BMI had an older age of disease onset. Results from linear regression analysis showed that with each increase of 1 BMI unit, the age of RRMS onset increases by 0.255 (95% CI 0.136 to 0.374) years, *p* < 0.001. CONCLUSIONS: Patients with higher BMI, as a parameter of MetS, exhibit increased age of RRMS onset. Our results may present an alternative personalized approach for diagnosis, prognosis, and/or prevention of RRMS.

## 1. Introduction

Multiple sclerosis (MS) is a complex chronic disease of the central nervous system (CNS) [1]. Although the exact etiopathogenesis of MS has not been well-established yet [2], there is accumulating evidence that demyelination, inflammation (perivascular and parenchymal), and neurodegeneration in the brain and the spinal cord are the major pathological processes present during the course of MS, and possibly cause the clinical manifestations [3]. The dissemination of the demyelination foci in time and space is a key feature of MS that has been encompassed in the diagnostic criteria [4], and it also adds to the heterogeneity of the symptomatology from patient to patient. The commonest form of MS is the relapsing-remitting form (RRMS), where the disease presents in relapses, with periods of complete or partial remission in between. The secondary progressive form (SPMS) represents a “progression” of RRMS, where no distinct relapses are usually noted, but a steady disease progression manifests instead. If it so from its onset, then it is named primary progressive (PPMS) [5]. Pathologically, the relapsing forms are more distinctively characterized by multifocal inflammation, whereas the progressive forms show more traits of chronic neuronal damage, which is reflected by the higher degree of disability they entail [6].

Epidemiologically, the prevalence of MS seems to vary geographically, and to increase with latitude; it is higher in countries of the northern hemisphere [7] and among developed countries [8]. It constitutes a main cause of neurological disability during adulthood, especially in younger adults [7].

In the multifactorial pathogenesis of MS, both genetic and non-genetic risk factors have been incriminated [9,10]. Its genetic background is known to be strong, with numerous susceptibility foci having been identified [11], and more than 110 genetic risk factors of MS have been pinpointed [12,13], though the genetic components alone do not suffice to explain its occurrence, as shown by the fact that homozygotic twins do not present the exact same rates of MS [14]. In fact, the genetic risk loci, alongside major risk HLA variants, can only explain approximately 30% of MS heritability, revealing the strong contribution of environmental factors to MS pathophysiology [15,16,17,18]. In this regard, various environmental factors have been pinpointed as risk factors for MS, such as viral infections [19], and interactions between specific polymorphisms and environmental risk factors have also been documented [20,21]. These joint effects and epigenetic mechanisms also appear to contribute, to some degree, to MS development and course [11,16].

Several environmental risk factors have been studied for their possible contribution to MS, such as vitamin D deficiency, smoking and other vascular risk factors, an Epstein-Barr Virus (EBV) infection, alongside obesity in childhood and early adulthood, and dietary habits, [2,15,22,23,24,25]. Regarding body weight and obesity, a recent meta-analysis reported lower mean body mass index (BMI) in patients with MS during the disease course compared to healthy individuals [26], increasing the evidence regarding the role of BMI and weight in MS [22,27,28].This is of particular interest, as increased BMI up until adolescence and childhood has been associated with MS, but there is no such indication for increased BMI during adulthood [22,27,28]. The importance of BMI is also evident through its impact on other neurodegenerative diseases, such as amyotrophic lateral sclerosis (ALS) and Parkinson’s disease (PD) [29,30,31].

In this regard, BMI is a component of the globally prevalent, metabolic syndrome (MetS) [32] which is defined as the concomitant manifestation of abdominal obesity, hyperlipidemia, hypertension, and insulin resistance [33]. Besides its implication in cardiovascular risk, it has been implicated in neurodegenerative disorders such as PD and Alzheimer’s disease (AD) [32,34], and limited data also indicate an impact of MetS in MS; some MetS components including BMI are related to MS disability [35,36]. Moreover, as in the case of MS, there is evidence of BMI-associated genetic susceptibility; genes in BMI genome-wide association studies loci are principally expressed in the CNS [37].

Overall, findings from studies regarding the role of BMI in MS remain contradictory so far. Moreover, there is relatively limited data and awareness regarding the BMI of Greek patients with MS. Furthermore, there is no relative information regarding a potential impact of BMI on the possible increased age of onset in patients with RRMS. In view of the former considerations, the objective of the present article is to present the results from an analysis regarding the relative role of BMI in MS in a Greek cohort. Patients with higher BMI exhibit increased age of RRMS onset. Our results may present an alternative personalized approach for diagnosis, prognosis, and/or prevention of RRMS.

## 2. Materials and Methods

### 2.1. Study Population

Data from Greek patients with RRMS were collected from the Papageorgiou General Hospital of Thessaloniki, a tertiary hospital located in North Greece. In total, data from 821 RRMS cases were collected for the study. Patients were considered eligible for the study if they had a diagnosis of clinically definite MS by the 2005 revised diagnostic McDonald criteria [38]. PPMS and SPMS were considered an exclusion criterion, so that our cohort would present phenotypic homogeneity. Participants’ sex, age at disease onset (defined as age at the appearance of the first neurologic symptom pertaining to MS), smoking, alcohol consumption, height, weight, and BMI were recorded. The study protocol received the approval of the Papageorgiou General Hospital of Thessaloniki Ethics Committee. The BMI was calculated as the weight in kilograms divided by the square of the height measured in meters [39].

### 2.2. Statistical Analysis

The descriptive statistics of the participants’ characteristics were expressed as mean and standard deviation (SD) for continuous variables, and as absolute values and/or percentages for categorical variables. The BMI values were also considered as quartiles, with Q1 corresponding to the lowest BMI. Comparisons for the demographic characteristics between the quartiles were made by Pearson’s chi-square test for the categorical variables and by ANOVA for the continuous variables. The calculation of the overall *p*-value was performed corresponding to trend for association. In case of a significant association, further post-hoc analysis was carried out, in order for differences in demographic characteristics between specific BMI quartiles groups to be identified.

Linear regression analyses (adjusted for sex, history of smoking, and history of alcohol consumption) were applied to explore the relationship between BMI (predictor) and age at onset of MS (outcome).

Statistical analyses were carried out with SPSS version 20.0 for Windows (SPSS Inc., Chicago, IL, USA). A *p*-value < 0.05 was named the threshold of statistical significance.

## 3. Results

A cohort of 821 (68.5% female, mean age of onset ± SD = 30.69 ± 9.44 years) patients with RRMS was included in the analysis. The demographic characteristics of the RRMS cohort are presented in Table 1.

Comparisons of participant characteristics by quartiles of BMI (with Pearson’s chi-square test for the categorical variables and ANOVA for the continuous variables) revealed that participants with the highest BMI had an older age of disease onset. The third and fourth BMI quartiles consisted of more male patients compared to other quartiles. Moreover, there was no difference between the BMI quartiles regarding history of smoking or alcohol consumption (Table 2).

Results from linear regression analysis revealed that BMI was correlated with age of RRMS onset. More precisely, with each increase of 1 BMI unit, the age of RRMS onset increases by 0.255 (95% CI 0.136 to 0.374) years, *p* < 0.001 (Table 3).

## 4. Discussion

In this study, we evaluated a considerable number of individuals with RRMS and assessed the relationship between BMI and the age of RRMS onset. We found that Greek patients with higher BMI have a RRMS onset significantly later than patients with lower BMI values. More specifically, we found that with each increase of 1 BMI unit, the age of onset is delayed for 0.255 years. The present study adds information to the existing bibliography on the role of BMI, as a parameter of MetS, in RRMS. In particular, we present additional data for RRMS in patients of Greek origin, adding to literature regarding southern Europe and the Caucasians.

The role of BMI in patients with MS has not been fully explained yet. Whereas a high BMI in adolescence and childhood has been associated with a higher risk of MS, there is no such indication for an increased BMI during adulthood [22,27,28]. BMI also seems to influence the presence of MS, as a few previous studies reported that patients with MS had significantly different BMI values (either lower or higher) compared to healthy controls [40,41].

Obesity, as a component of MetS [42], is among the factors that potentially influence autoimmunity and is connected to many diseases, including MS [43]. However, the rates of obesity and its contribution to MS vary among populations with different origins [44], and as previously noted, between patients and healthy controls. As such, the relationship between BMI and MS needs to be carefully examined, as this variation could reflect other pathological processes. For example, these differences could be related to nutrition, since MS patients may be riddled by dysphagia [45], or even depression [46], which is known to impact alimentation habits [47]. Additionally, this variation could be attributed to the extent of disability, as reduced independence can lead to reduced sustenance of patients with MS during the disease course. Finally, it could be the result of the direct genetic component of BMI [26,48]. As it is thus evident, more data in pinpointing the origins of the relationship between body weight and MS are needed.

Obesity, and therefore BMI, is also reasonably influenced by dietary patterns. Recently, an Iranian study found that following a Mediterranean diet is linked to a decreased MS risk [49], and other diets also appear to have an effect on MS [50,51,52]. Likewise, recent evidence indicates that fasting alleviates both clinical and pathological indications of MS [53]. Fasting also inhibits and/or effectively reduces obesity indices (BMI, body weight, waist circumference, body fat mass) and other parameters/comorbidities of MetS, which represents a significant risk factor for several other neurodegenerative disorders including MS, AD, and PD [53,54,55]. On the contrary, metabolic dysfunction owing to high fat diet (HFD) may contribute to the initiation, progression, or disability load of MS [15,56]; HFD-associated alterations in metabolic regulators lead to mitochondrial dysfunction, oxidative stress, and loss of oligodendrocyte and myelinating cells occurring in MS [54,57].

Moreover, BMI may be influenced by the gut microbiota. Based on the “gut-brain axis” hypothesis [58], gut microbiota are heavily involved in the development of the immune system and are thus implicated in various immune mechanisms, including the emergence of autoreactive CD4+ cells, antigenic mimicry, mainly with oligodendrocytes and the myelin glycoprotein, or pathways of the innate immune system [59]. Likewise, gut microbiota have been linked to MetS components, and MetS occurs more often in patients with small intestinal bacterial overgrowth (SIBO) [32]. In this respect, SIBO is highly predominant in patients with MS, thereby possibly playing a potential role in MS pathophysiology [60]. Additionally, reports of gut microbiota composition differences between MS patients and healthy individuals have emerged [61,62], and specific pathways of the interaction between microbiota and neuroinflammation have started to be elucidated [63]. Differences in microbiota composition have also been reported for obese and lean individuals [64], and the microbiome of obese people has been reported to increase the caloric intake from nutrition [65]. This is very important, considering how obesity has been repeatedly tied to increased risk of developing autoimmune disorders and to worse clinical courses [43].

This represents a very interesting novel research field, since the microbiome is considered a “druggable” target [66], and interventions on it may influence BMI and MS parameters. In this sense, caloric restriction clearly influences the immune system and the courses of autoimmune disorders [67,68]. Intermittent fasting was shown to confer benefits in experimental autoimmune encephalomyelitis, the animal model of MS, by modifying gut microbiota and enhancing anti-oxidant pathways and the survival of regulatory T-cells [25,69]. These results could not be replicated for a small cohort of MS patients that underwent intermittent fasting [69], though a fasting-mimicking diet was shown to lead to better quality of life in MS patients [70]. Despite the preliminary results, dieting interventions merit more research, especially if there is evidence to support their various benefits in MS [25]. In this line of thought, novel interventions are urgently needed in the treatment of autoimmune diseases, MS included, since traditional pharmacological treatments are not wholly efficient and can lead to several side-effects. Non-pharmacological interventions have started to gain ground in alleviating symptomatology [71], and alternatives that may shift the very pathogenic processes of neuroinflammation, such as possibly those involved with BMI and the gut-brain axis, will hold a lot of potential as well.

Moving on, BMI may also have an effect on vitamin D levels and T-regulatory cells (Tregs) functions [72], factors also shown to be involved in MS. In this regard, for instance, Tregs are significantly reduced in children with MetS, and their reduced number or function plays a role in low-grade inflammatory process linked with obesity [73]. Likewise, disturbances in Tregs gene expression are observed in children with MetS [74]. Therefore, the introduction or restoration of functional Treg cells in children with MetS might be introduced in the design of therapeutic strategies in MetS-related obesity associated with immunologic disorders such as MS [73]. Several autoimmune and inflammatory pathologies in humans are associated with functional defects in Tregs, whereas adoptively transferred Tregs limit the pathogenicity in animal models of autoimmunity. Thus, Tregs represent a promising stage for effective adoptive immunotherapy of chronic inflammatory autoimmune disorders, including multiple sclerosis [75].

As in the case of our RRMS patients, MetS seems to be more prevalent in women, and its prevalence increases with age [76]. Moreover, beyond obesity, MS is related to additional components and/or comorbidities of MetS such as type 2 diabetes mellitus, insulin resistance, hypertension, dyslipidemia, or cardiovascular disease [35,77,78]. Therefore, it is possible that MS patients with MetS components and/or comorbidities may exhibit increased age of RRMS onset, and thus further studies are needed. Additionally, a later disease onset has been tied to a worse prognosis of MS, as expressed by higher disability [79], and also seems to be a poor prognosis factor independent of the initial disease course [80]. This may also hint towards an interaction between MetS, which becomes more prevalent with age, and MS prognosis. In a recent study, both older age and higher BMI values were associated with slower walking speed and higher global disability in MS patients [81]. A BMI of value higher than 30, paired with a smoking, was further associated with conversion from RRMS to SPMS in a large cohort of MS patients [56]. This line of studies linking obesity/higher BMI to worse prognosis showcase the importance of encompassing BMI when assessing MS patients, and possibly highlight the need of a more aggressive, earlier intervention.

At this point, some limitations of the current study need to be addressed. Our study is a retrospective analysis of prospectively collected data, therefore information bias may be present. Moreover, our results would have had more robustness if other predictors, such as a history of EBV infection, vitamin D serum levels, dietary habits, genetic loci, and other vascular risk factors [82,83,84,85,86,87] had been included in the regression models. Furthermore, with exception of BMI and smoking habit, we did not estimate additional MetS components and/or comorbidities. Finally, analysis according to MS disability as expressed with expanded disability status scale (EDSS) or multiple sclerosis severity score (MSSS) [88,89] would have let us produce even more accurate conclusions.

The current study also possesses certain strengths. The clinical evaluation was conducted by a senior neurologist with an expertise in multiple sclerosis, so that the diagnosis of RRMS could be rigorously assessed. Additionally, our sample was sufficiently large. Finally, the RRMS cohort was homogenous, as the participants came from the same area (Northern Greece) and were monitored at the same hospital.

## 5. Conclusions

In conclusion, this study demonstrated that BMI, as a MetS component, was linked to the age of RRMS onset. More precisely, we found that patients with higher BMI present an increased age of RRMS onset. In view of the retrospective design of the study, our results should be replicated in multi-ethnic prospective studies as well. Moreover, the role of BMI and other MetS parameters should be analyzed pertaining to other MS endophenotypes, such as neurologic deficits or disability progression, since there is evidence to suggest its implication in worse prognoses. Finally, longitudinal studies examining how BMI may impact the magnitude of relapse or remission, or migration to progressive state are of great importance. Bearing in mind the increased cost of MS management and the life expectancy of patients, this study represents an effort to offer a personalized approach to each patient, regarding diagnosis, prognosis, and/or prevention of RRMS.

## Figures and Tables

**Table 1:** Demographic characteristics of patients with RRMS (n = 821).

Characteristics	
Age at onset, years (mean ± SD)	30.69 ± 9.44
Sex (female) n (%)	562 (68.5%)
History smoking	
Yes, %	69.9%
No, %	30.1%
History of alcohol consumption	
Yes, %	13.9%
No, %	86.1%
BMI (mean ± SD)	25.72 ± 6.21
Height, m (mean ± SD)	1.69 ± 0.084
Weight, kg (mean ± SD)	71.97 ± 15.53

RRMS, Relapsing-remitting multiple sclerosis; SD, standard deviation; BMI, body mass index.

**Table 2:** Demographic characteristics of RRMS patients by quartiles of BMI.

	1st Quartile, Score: 15.92 to 21.53244 (n = 205)	2nd Quartile, Score: >21.53244 to 24.22145 (n = 206)	3rd Quartile, Score: > 24.22145 to 28.07073 (n = 205)	4th Quartile, Score: >28.07073 to 66.14 (n = 205)	*p* for Trend
Age at onset, years (mean ± SD)	27.91 ± 7.92 ^c, d^	29.72 ± 9.39 ^c, d^	32.36 ± 10.17 ^a, b^	32.90 ± 9.38 ^a, b^	**<0.001**
Sex (female) n (%)	182 (88.8%) ^b, c, d^	148 (78.1%) ^a, c, d^	106 (51.7%) ^a, b, d^	126 (61.5%) ^a, b, c^	**0.013**
History smoking					0.930
Yes (%)	71.9%	66.9%	70.7%	70.2%	
No (%)	28.1%	33.1%	29.3%	29.8%	
History of alcohol consumption					0.401
Yes (%)	12.1%	11.9%	20.5%	11.9%	
No (%)	87.9%	88.1%	79.5%	88.1%	

RRMS, Relapsing-remitting multiple sclerosis; BMI, body mass index. ^a, b, c, d^ indicate statistically significant difference compared to value in the 1st, 2nd, 3rd, and 4th quartiles, respectively. Statistically significant values are given in bold.

**Table 3:** Linear regression analyses for association between age at MS onset with BMI.

	Beta ± SE	95%CI	*p*-Value
Unadjusted	0.255 ± 0.061	(0.136 to 0.374)	**<0.001**
Adjusted *	0.273 ± 0.061	(0.152 to 0.393)	**<0.001**

SE, standard error; CI, confidence interval. * Adjusted for sex, history of smoking, and history of alcohol consumption. Statistically significant values are given in bold.

## Data Availability

The data presented in this study are available on request from the corresponding author.
